# Infection by Anaplasma phagocytophilum Requires Recruitment of Low-Density Lipoprotein Cholesterol by Flotillins

**DOI:** 10.1128/mBio.02783-18

**Published:** 2019-03-26

**Authors:** Qingming Xiong, Mingqun Lin, Weiyan Huang, Yasuko Rikihisa

**Affiliations:** a Department of Veterinary Biosciences, The Ohio State University, Columbus, Ohio, USA; University of Illinois at Chicago

**Keywords:** *Anaplasma phagocytophilum*, cholesterol, flotillin-1, flotillin-2, LDL, acid lipase

## Abstract

Anaplasma phagocytophilum is an obligatory intracellular bacterium that proliferates in membrane-bound inclusions. A. phagocytophilum is dependent on cholesterol and acquire cholesterol from low-density lipoprotein (LDL) endocytosed by mammalian host cells. The mechanism of cholesterol transport to Anaplasma inclusions, however, is not fully understood. Flotillin-1 (FLOT1) and FLOT2 are cholesterol-associated membrane proteins that form a heterodimer and/or oligomer complex. Here, we found that Anaplasma infection was significantly reduced by small interfering RNA (siRNA) knockdown of FLOT1 or FLOT2. Anaplasma inclusions were encircled with small vesicles containing endogenous FLOT1 or FLOT2 or with ectopically expressed FLOT1-mCherry and FLOT2-green fluorescent protein (FLOT2-GFP). FLOT1- and FLOT2-containing vesicles were enriched with unesterified cholesterol, as indicated by labeling with filipin and aminomethyl coumarin acetic acid-conjugated theonellamide. Localization of FLOT2 to Anaplasma inclusions was dependent on cholesterol, as FLOT2-GFP bearing two mutations in the cholesterol recognition/interaction motif could not target the inclusions. The cholesterol-sequestering agent methyl-β-cyclodextrin abrogated FLOT1 localization to Anaplasma inclusions and cleared infection. FLOT2-GFP also localized to fluorescent 3,3′-dioctadecylindocarbocyanine (DiI)-LDL-containing vesicles, including those surrounding Anaplasma inclusions. FLOT2 siRNA knockdown blocked DiI-LDL trafficking to Anaplasma inclusions and reduced bacteria-associated cholesterol amount, and therefore inhibiting Anaplasma infection. Vesicles containing acid lipase, which hydrolyzes LDL cholesterol esters to free cholesterol, colocalized with FLOT2 and encircled Anaplasma inclusions, while the acid lipase inhibitor orlistat significantly inhibited Anaplasma replication. Together, the data revealed that FLOTs are crucial for Anaplasma replication in host cells, likely by aiding vesicular traffic of LDL-derived free cholesterol to Anaplasma inclusions, and suggest a new way of inhibiting Anaplasma infection.

## Abstract (precis)

Cholesterol is essential for animal cells, but most bacteria do not depend on cholesterol and instead lack cholesterol. However, the intracellular Gram-negative bacterium Anaplasma phagocytophilum that causes human granulocytic anaplasmosis (HGA) is unusual, as it contains significant amount of cholesterol and depends on cholesterol for survival and infection. A. phagocytophilum lacks genes for cholesterol biosynthesis or modification but acquire cholesterol from host cells exclusively from the LDL uptake pathway by a yet-to-be defined mechanism. Here, we uncovered a role of cholesterol-binding proteins FLOT1 and FLOT2 in LDL-derived cholesterol trafficking to Anaplasma inclusions and cholesterol acquisition by Anaplasma species. Importantly, we found that FLOTs localize to A. phagocytophilum-containing inclusions and the compartments containing LDL, and the acid lipase inhibitor orlistat significantly inhibits Anaplasma replication. Our data suggest a fundamental role of FLOTs in intracellular vesicular transport of LDL-derived free cholesterol and may provide insight regarding a new therapeutic target for HGA treatment.

## INTRODUCTION

Anaplasma phagocytophilum is a causative agent of human granulocytic anaplasmosis (HGA), a major tick-borne zoonosis emerging in the United States and other parts of the world (1). HGA is an acute febrile disease and is potentially fatal, especially in elderly or immunocompromised individuals (2). A. phagocytophilum is an obligatory intracellular bacterium that proliferates in membrane-bound inclusions (called morulae) in neutrophils and endothelial cells in mammalian hosts (3, 4). During infection progression, clusters of bacterial inclusions expand to occupy most of the cytoplasm of infected cells (1). A. phagocytophilum inclusions serve as a unique niche for A. phagocytophilum to establish its infection and allow acquisition of nutrients and replication in seclusion from lysosomes and NADPH oxidase (1, 5, 6).

A. phagocytophilum is a unique Gram-negative bacterium because it contains a substantial amount of cholesterol in its membrane, and cholesterol is essential for its survival *in vitro* (7). In mice, high blood cholesterol causes more severe clinical signs with a 10-fold higher bacterial load in the blood than with normal cholesterol levels (8). A. phagocytophilum upregulates and accumulates low-density lipoprotein (LDL)-containing vesicles around bacterial inclusions and hijacks the vesicular cholesterol transport via Niemann-Pick type C 1 (NPC1) vesicles that are free from late-endosome/lysosomal markers (9, 10). In addition, cholesterol-enriched lipid rafts on host cells play a critical role in A. phagocytophilum internalization and infection of host cells (11).

Flotillins (FLOTs) are well conserved (from bacteria to mammals [12]) membrane-associated proteins that belong to a large family of proteins containing a stomatin-prohibitin-flotillin-HflK/C (SPFH) domain (13). FLOTs in mammalian cells are involved in several cellular processes, including endocytosis, membrane trafficking, signaling, and scaffolding functions (14). Their two isoforms, FLOT1 and FLOT2 (reggie-2 and reggie-1, respectively), have a molecular mass of ∼48 kDa and form a heterodimer and/or oligomer complex, and their amino acid sequences show about 50% identity (15). Thus, it is expected that FLOT1 and FLOT2 share similar functions. Both FLOT1 and FLOT2 are expressed and colocalized in human neutrophils (16). It has been shown that an increase in cellular cholesterol leads to the intracellular vesicular translocation of FLOT2 from the plasma membrane and, reciprocally, cholesterol depletion drives vesicular FLOT2 to the plasma membrane (17). FLOT1 and FLOT2 have 1 and 2, respectively, cholesterol recognition/interaction amino acid cholesterol-binding domains [CRAC; L/V-(X)(1-5)-Y-(X)(1-5)-R/K] (18). This CRAC domain largely determines the translocation of FLOT2 from the plasma membrane pool to the subcellular vesicular pool (19). FLOT2-green fluorescent protein (FLOT2-GFP) partially localizes to LysoTracker-positive acidic compartments and multivesicular bodies, but it is not involved in the endocytosis of epidermal growth factor and glycosylphosphatidylinositol (GPI)-anchored proteins (17). FLOT1 and FLOT2 are independent from clathrin (17) or caveolin (20–23). FLOT1 was found to be present on the membranes of vacuoles containing Brucella abortus, Coxiella burnetii, Mycobacterium marinum, and Chlamydophila pneumoniae (24–27).

Here, we examined the involvement and role of FLOT1 and FLOT2 in A. phagocytophilum infection, particularly LDL-derived cholesterol trafficking to Anaplasma inclusions and cholesterol acquisition by Anaplasma species. We demonstrate that FLOT1 and FLOT2 vesicles are accumulated on A. phagocytophilum inclusions, contain nonesterified cholesterol, and colocalize with LDL-containing compartments and with acid lipase that is known to hydrolyze cholesterol ester in LDL to liberate free cholesterol (28). Moreover, our data reveal that FLOT1 and FLOT2 play a crucial role in A. phagocytophilum infection and cholesterol acquisition.

## RESULTS

### A. phagocytophilum infection is dependent on FLOT1 and FLOT2.

**FIG 1:**
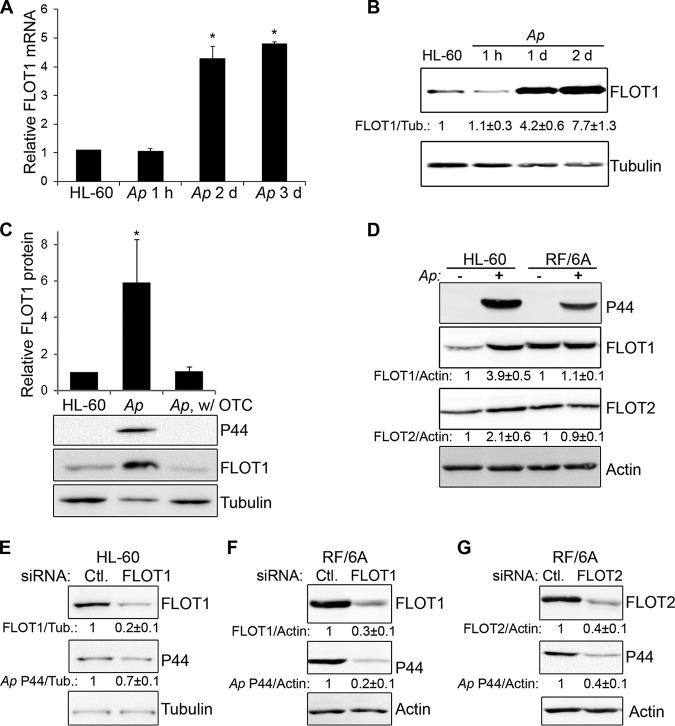
FLOTs are required for *A. phagocytophilum* infection. (A) FLOT1 mRNA levels in uninfected HL-60 and *Anaplasma* (*Ap*)-infected HL-60 cells at 1 h, 2 days (d), and 3 days postinfection (dpi) normalized to human G3PDH mRNA with the ratio of HL-60 cell sample arbitrarily set as 1. The results are presented as the mean ± standard deviation from three independent experiments. The asterisk indicates a significant difference compared with control by analysis of variance (*P < *0.05). (B) FLOT1 protein levels in uninfected HL-60 and *Anaplasma*-infected HL-60 cells at the indicated time points (1 hpi, 1 dpi, and 2 dpi) were assessed by Western blotting using anti-FLOT1 monoclonal antibody with α-tubulin as the normalization control. Density ratios of FLOT1/tubulin (Tub.) intensities were determined by densitometry, with the ratio of that from HL-60 cell sample arbitrarily set as 1. The results are representative of three independent experiments. (C) *Anaplasma*-infected HL-60 cells at 1 dpi were treated with oxytetracycline (OTC; 10 µg/ml) for 24 h. FLOT1 levels were assessed by Western blotting using the anti-FLOT1 antibody. The bacterial load was determined by assessing the levels of P44 using the antibody 5C11, with α-tubulin serving as the normalization control. The results are representative of three independent experiments. The asterisk indicates a significant difference compared with FLOT1 protein level in HL-60 cells by analysis of variance (*P < *0.05). (D) FLOT1 and FLOT2 protein levels in uninfected and *Anaplasma*-infected HL-60 cells at 2 dpi and RF/6A cells at 3 dpi were assessed by Western blotting using the anti-FLOT1 and FLOT2 antibodies. The bacterial load was determined by assessing the P44 levels using the 5C11 monoclonal antibody, with actin serving as the normalization control. Density ratios of FLOT1 and FLOT2 versus actin intensities were determined by densitometry, with the ratios of those from uninfected cell samples arbitrarily set as 1. The results are representative of three independent experiments. (E to G) HL-60 (E) or RF/6A (F and G) cells were transfected with FLOT1 (D and E), FLOT2 (F), or control (Ctl.) siRNA. At 1 dpt, *A. phagocytophilum* was added to the cultures, and the cells were then cultured for 2 days. The cell samples were lysed and subjected to Western blotting using antibodies against FLOT1 and FLOT2 and P44. Tub. or actin served as the normalization control. The amounts of FLOT1 and FLOT2 and P44 (relative to actin or Tub.) were determined by densitometry. The values under the bands show the relative ratios of band intensities, with the ratios of those from samples transfected with control siRNA arbitrarily set as 1. The results are representative of four independent experiments.

Expression levels of FLOT1 and FLOT2 during A. phagocytophilum infection in human promyelocytic leukemia (HL-60) and monkey endothelial (RF/6A) cell lines were measured by quantitative reverse transcription-PCR (qRT-PCR) and Western blotting. Levels of Anaplasma infection in the protein samples were shown by Western blotting using anti-P44 (the major outer membrane protein of A. phagocytophilum) (29, 30). Compared with uninfected HL-60 cells, FLOT1 was significantly upregulated in Anaplasma-infected HL-60 cells at mRNA (Fig. 1A) and protein (Fig. 1B) levels. The upregulation of FLOT1 was dependent on bacterial protein synthesis because treatment with the antibiotic oxytetracycline at 1 day postinfection (dpi) completely abolished the infection and upregulation of FLOT1 at 2 dpi (Fig. 1C). Compared with FLOT1, levels of FLOT2 upregulation in Anaplasma*-*infected HL-60 cells were lower (Fig. 1D). In contrast to HL-60 cells, no significant increase in FLOT1 or FLOT2 levels was found in Anaplasma*-*infected RF/6A cells (Fig. 1D). FLOT1 and FLOT2 levels are cell type and tissue specific (31, 32). We found that the relative amounts of FLOT1 and FLOT2, especially FLOT1, in RF/6A cells were greater than those in HL-60 cells (Fig. 1D). Indeed, uninfected RF/6A cells had high preexisting FLOT levels comparable to those of Anaplasma-infected HL-60 cells (Fig. 1D).

We next examined whether FLOT1 and FLOT2 are required for Anaplasma infection. FLOT1 was knocked down in HL-60 and RF/6A cells by small interfering RNA (siRNA) transfection. The effectiveness of knockdown in FLOT1 siRNA- and control siRNA-treated cells was determined by examining FLOT1 protein levels using Western blotting. Since FLOT1 and FLOT2 are known to form stable tetramers in membrane microdomains, the expression of FLOT1 and FLOT2 is interdependent, and downregulation of one FLOT reduces the stability of the other (33–35). In agreement with these reports, our data showed that FLOT2 siRNA significantly reduced the protein amounts of both FLOT2 and FLOT1 (over 60%), whereas FLOT1 siRNA reduced the protein amount of FLOT1 significantly but of FLOT2 to a lesser extent (>30%; see Fig. S1 in the supplemental material). Treatment with FLOT1 siRNA for 2 days reduced its protein level in HL-60 and RF/6A cells by over 70%, and Anaplasma infection as represented by P44 levels in both HL-60 (Fig. 1E) and RF/6A cells (Fig. 1F) was significantly reduced. Anaplasma infection was also significantly reduced in FLOT2 siRNA-treated (for 2 days) RF/6A cells (Fig. 1G). These data demonstrate that A. phagocytophilum upregulates FLOTs (at least in HL-60 cells), and infection is reduced by siRNA knockdown of FLOT1 or FLOT2.

10.1128/mBio.02783-18.1 FIG S1 Expression of FLOT1 and FLOT2 is interdependent. RF/6A cells were transfected with scrambled control (CTL) siRNAs, or siRNAs against FLOT1, FLOT2, or FLOT1 and FLOT2. At 2 dpt, the cell samples were lysed and subjected to Western blotting using antibodies against FLOT1/2 and actin, which was served as the protein loading control. The amount of FLOT1 and FLOT2 (relative to actin) was quantitated by densitometry, and the values under the bands show the relative ratios of band intensities, with the ratios of those from samples transfected with control siRNA arbitrarily set as 1. Images were one representative, and the ratios were the average from three independent experiments. Download FIG S1, TIF file, 1.4 MB. Copyright © 2019 Xiong et al. 2019 Xiong et al. This content is distributed under the terms of the Creative Commons Attribution 4.0 International license.

### A. phagocytophilum growth, but not internalization into host cells, is dependent on FLOT1 and FLOT2.

**FIG 2:**
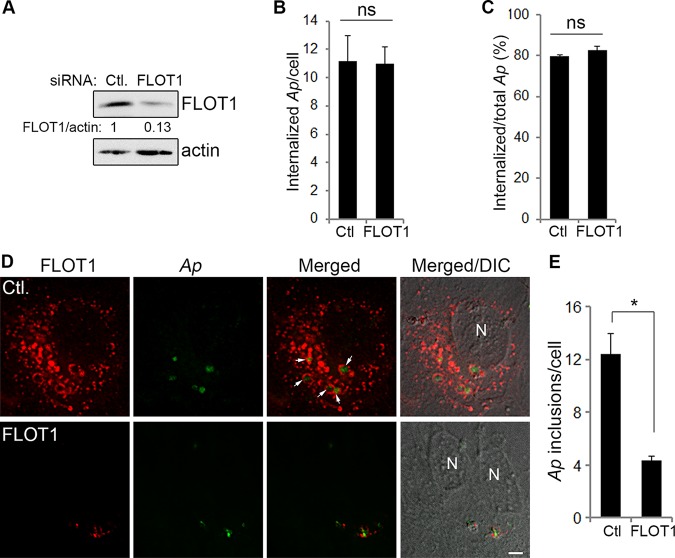
FLOT1 is required for the growth but not internalization of *A. phagocytophilum* into host cells. (A) RF/6A cells were transfected with FLOT1 and control (Ctl) siRNA, and relative FLOT1 levels in transfected cells at 2 dpt were determined by Western blotting using anti-FLOT1 antibody, with actin as the normalization control. The amounts of FLOT1 (relative to actin) were determined by densitometry. The values under the bands show the relative ratios of band intensities, with the ratios of those from samples transfected with control siRNA arbitrarily set as 1. The result is the representative of three independent experiments, similar to Fig. 1F. (B and C) Host cell-free *A. phagocytophilum* (*Ap*) was added into transfected cell cultures at 2 dpt and incubated for 2 h to allow internalization. The cells were then harvested and subjected to two-step sequential immunofluorescence labeling of external and total bacteria using anti-P44 without and with permeabilization, respectively, to determine the proportion of internalized *A. phagocytophilum*. The number of internalized *A. phagocytophilum* per cell (B) and the percentage of internalized *A. phagocytophilum* in total bacteria (C) were scored in >200 cells. The results are presented as the mean ± standard deviation from three independent experiments. ns, no significant difference. (D and E) Host cell-free *A. phagocytophilum* was added to FLOT1 and Ctl siRNA-transfected cells at 2 dpt and then cultured for 1 day. At 1 dpi/3 dpt, the cells were harvested, stained with rabbit anti-Asp62 (green) and mouse anti-FLOT1 (red), and visualized by fluorescence microscopy. White arrows indicate *Anaplasma* inclusions encircled with FLOT1. N, nucleus. Merged/DIC, merged image with differential interference contrast. The data shown are representative of at least three independent experiments. Scale bar = 5 µm (D). The bacterial load was scored in >200 cells (E). The results are presented as the mean ± standard deviation from three independent experiments. *, *P < *0.05.

FLOTs are associated with endocytosis of a growing list of molecules, including GPI-anchored proteins, cholera toxin B subunit, Niemann-Pick C1-like 1 (NPC1L1) protein, amyloid precursor protein, cationic molecules, polyplexes, proteoglycans, and proteoglycan-bound ligands, although the mechanisms by which FLOTs function in endocytosis are still unclear (36, 37). Our previous work showed that A. phagocytophilum enters host cells through receptor-mediated endocytosis in a clathrin-independent mechanism (11). Given the requirement of FLOT1 and FLOT2 in Anaplasma infection (Fig. 1E to G), we examined whether FLOTs are involved in the internalization of bacteria into the host cells or in the growth stage in the cells. RF/6A cells were treated with FLOT1 siRNA for 2 days, which greatly reduced FLOT1 compared to negative-control siRNA (Fig. 2A), and then were incubated with host cell-free A. phagocytophilum for 2 h to allow internalization. As PCR or Western immunoblot analysis cannot distinguish bound versus internalized bacteria, we carried out two-step sequential immunofluorescence labeling of external and total bacteria (38) to determine the proportion of internalized A. phagocytophilum. Our quantitative data showed there was no significant difference in the numbers of internalized Anaplasma bacteria/cell or the percentage of internalized/total Anaplasma bacteria at 2 hours postinfection (hpi) between FLOT1 siRNA and control siRNA-treated cells (Fig. 2B and C). Similar results were obtained from FLOT2 siRNA-treated cells (Fig. S2A to C). However, at 3 days posttransfection (dpt) with siRNA and 1 dpi, the number of Anaplasma inclusions per cell was significantly reduced in FLOT1 siRNA-treated cells compared with control siRNA-treated cells (Fig. 2D and E). Since Anaplasma infection as represented by the P44 protein amount was significantly reduced by FLOT1 and FLOT2 siRNA treatment (Fig. 1E to G), collectively, these data indicate that FLOT1 and FLOT2 are critical for bacterial intracellular growth rather than the entry step. Our data also suggest that A. phagocytophilum enters host cells through FLOT-independent endocytosis.

10.1128/mBio.02783-18.2 FIG S2 FLOTs were not required for *A. phagocytophilum* internalization. (A) RF/6A cells were transfected with FLOT2 and control (Ctl.) siRNAs, and FLOT2 levels in transfected cells at 2 dpt were determined by Western blotting using anti-FLOT2 antibody with actin as the normalization control. The amounts of FLOT2 (relative to actin) were determined by densitometry with the ratio of that from sample transfected with control siRNA arbitrarily set as 1. The result shown is representative of three independent experiments similar to Fig. S1. (B and C) Host cell-free *A. phagocytophilum* was added into FLOT2 and control (Ctl.) siRNAs-transfected cells. At 2 hpi, the cells were harvested, and two-step labeling was performed using anti-P44 (5C11) and visualized by DeltaVision deconvolution microscopy. The number of internalized *A. phagocytophilum* per cell (B) and the percentage of internalized *Anaplasma* per total bacteria (C) were scored in >200 cells. The data shown are representative of at least three independent experiments. *Ap, Anaplasma*; ns, no significant difference. Scale bar = 5 µm. Download FIG S2, TIF file, 0.2 MB. Copyright © 2019 Xiong et al. 2019 Xiong et al. This content is distributed under the terms of the Creative Commons Attribution 4.0 International license.

### FLOT vesicles surround A. phagocytophilum inclusions.

**FIG 3:**
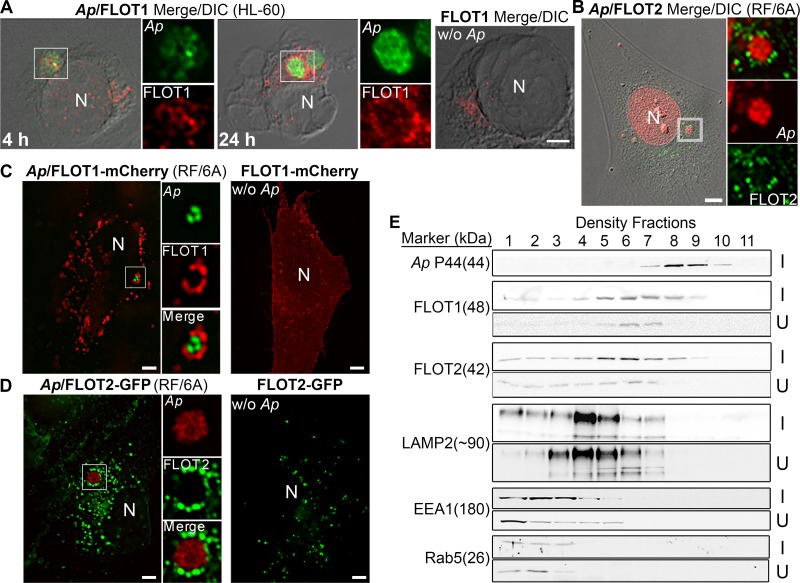
FLOT1 and FLOT2 encircle *A. phagocytophilum* inclusions. (A) Uninfected (without *Ap*) and *Anaplasma*-infected (*Ap*) HL-60 cells at 4 and 24 hpi were fixed, labeled with anti-Asp62 (*Ap*, green), and mouse anti-FLOT1 (red). Merge/DIC, merged image with differential interference contrast. (B) *Anaplasma*-infected RF/6A cells at 2 dpi were fixed and labeled with mouse anti-FLOT2. Nucleus (N) and *A. phagocytophilum* were labeled by DAPI (pseudocolored in red). (C and D) FLOT1-mCherry (C) and FLOT2-GFP (D) plasmids were transfected into *Anaplasma*-infected and uninfected RF/6A cells at ∼1 dpi. At 1 dpt (2 dpi), RF/6A cells were fixed, labeled with anti-*Ap* P44 (5C11) (*Ap*, green in panel C and red in panel D). Fluorescence images were visualized and captured by a DeltaVision deconvolution fluorescence microscope, and the figures in panels A to D are representative of at least three independent experiments. N, nucleus. Scale bar = 5 µm. The boxed areas were enlarged to the right. (E) *Anaplasma*-infected (I) and uninfected (U) HL-60 cells were fractionated by density gradient ultracentrifugation (5 to 25% OptiPrep). An equal volume from each fraction (from 1 to 11) was subjected to Western blotting using FLOT1 and FLOT2 antibodies. The molecular weights (in kilodaltons) for each protein are shown in parentheses. Anti-P44 was used to identify fractions containing *Anaplasma* inclusions. The results shown are representative of three independent experiments.

FLOTs are found in various cellular locations, including the plasma membrane, intracellular organelles, and even the nucleus (39). Since FLOTs are required for Anaplasma intracellular growth, we examined the cellular distribution of endogenous FLOT1 and FLOT2 in Anaplasma-infected and uninfected HL-60 and RF/6A cells by double-fluorescence labeling to detect FLOTs and A. phagocytophilum. More and larger FLOT1 puncta were found in Anaplasma-infected HL-60 cells than in uninfected cells (Fig. 3A). Notably, larger FLOT1 and FLOT2 puncta were enriched around Anaplasma inclusions, although they were also present in other areas in the cells (Fig. 2D, arrows, and Fig. 3A and B). The accumulation of FLOT1 puncta on Anaplasma inclusions was observed as early as 4 hpi (Fig. 3A). Localization patterns of FLOT1 and FLOT2 were more clearly observed in Anaplasma-infected RF/6A cells (Fig. 3B and S3A and B) because these endothelial cells are thinly spread and adherent, whereas HL-60 cells are round, thicker, and nonadherent. There were no cross-reactions between rabbit IgG and anti-mouse secondary antibodies or between mouse IgG and anti-rabbit secondary antibodies, as shown using normal rabbit and mouse IgGs as controls (Fig. S3C).

10.1128/mBio.02783-18.3 FIG S3 FLOT1 encircles *A. phagocytophilum* inclusions in infected cells. (A) *A. phagocytophilum*-infected and uninfected (w/o *Ap*) RF/6A cells were fixed, labeled with anti-Asp62 (*Ap,* green) and anti-FLOT1 (red), and visualized by DeltaVision deconvolution microscopy. N, nucleus. Merged/DIC, image merged with differential interference contrast. Scale bar = 5 µm. The results are representative of at least four independent experiments. (B) *Anaplasma*-infected RF/6A cells were fixed at 2 dpi, labeled with DAPI (blue and grey pseudocolor) and anti-FLOT1 (red), and visualized by DeltaVision deconvolution microscopy. Scale bar = 5 µm. The images shown are representative of at least two independent experiments. (C) *Anaplasma*-infected HL-60 cells were stained with normal rabbit IgG (green) and anti-FLOT1 (red) (left; *Anaplasma* negative control) or with anti-Asp62 (green) and normal mouse IgG (red) (right; FLOT1 negative control) and visualized by DeltaVision deconvolution microscopy. The results shown are representative of at least three independent experiments. Scale bar = 5 µm. Download FIG S3, TIF file, 1.9 MB. Copyright © 2019 Xiong et al. 2019 Xiong et al. This content is distributed under the terms of the Creative Commons Attribution 4.0 International license.

We next examined whether ectopically expressed FLOT1-mCherry or FLOT2-GFP can localize to Anaplasma inclusions in transfected RF/6A cells. FLOT1-mCherry and FLOT2-GFP localized as punctate patterns surrounding Anaplasma inclusions (Fig. 3C and D). At 2 dpi, most of Anaplasma inclusions were encircled by FLOT1-mCherry- and FLOT2-GFP-containing vesicles in RF/6A cells. Together, these data revealed that FLOT1 and FLOT2 form more and larger vesicles in infected cells than in uninfected cells and are recruited to Anaplasma inclusions in these mammalian host cells.

We further fractionated organelles by OptiPrep density centrifugation to analyze the distribution of endogenous FLOT1 and FLOT2 and to analyze Anaplasma inclusions. This method keeps Anaplasma inclusions intact, as previously shown (5, 10, 40). Western blotting of the resulting fractions showed that FLOT1 and FLOT2 were upregulated, shifted to higher-density fractions, and partially cofractionated with Anaplasma inclusion fractions (no. 7 to 10) in infected cells compared to uninfected cells (Fig. 3E). Most of lysosome-associated membrane protein 2 (LAMP2; a marker of late endosomes and lysosomes) and all early endosome antigen 1 (EEA1) and Rab5 (markers of early endosomes) did not cofractionate with Anaplasma inclusions. Taken together, these results indicate that FLOT1 and FLOT2 are recruited to Anaplasma inclusions independent of endosomes or lysosomes.

### FLOT vesicles are enriched in unesterified cholesterol.

**FIG 4:**
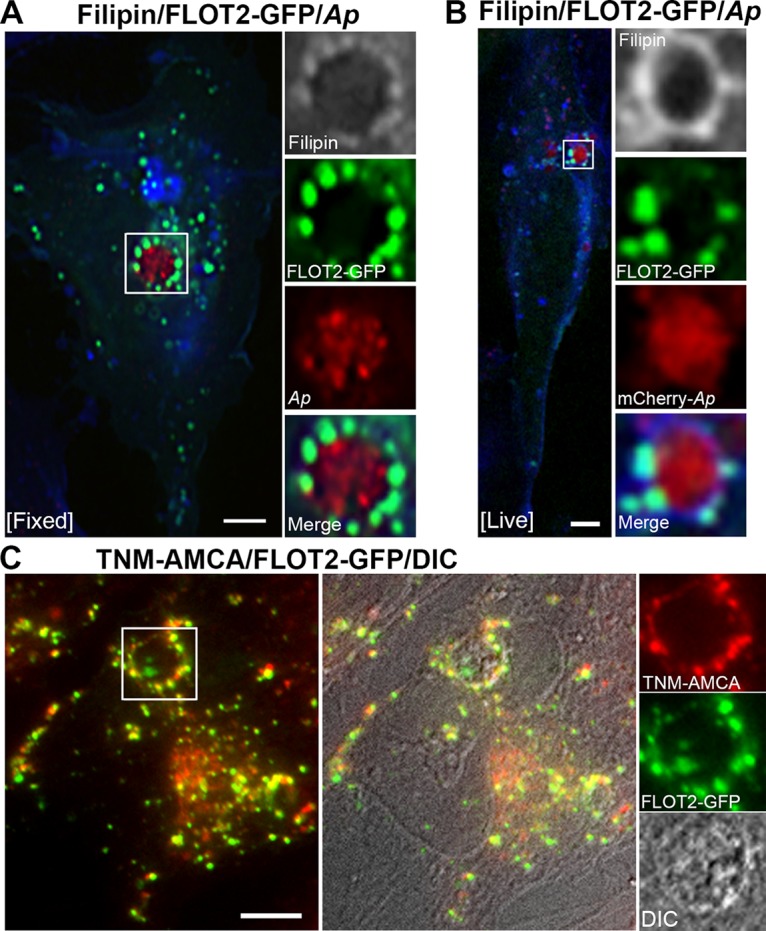
FLOT2-positive vesicles are enriched in free cholesterol. (A and B) FLOT2-GFP plasmid was transfected into *A. phagocytophilum*-infected (A) or mCherry-*A. phagocytophilum*-infected (B) RF/6A cells at ∼1 dpi. At 1 dpt (2 dpi), RF/6A cells were fixed, labeled with anti-P44 (*Ap*) and filipin (blue) (A) or live cells were incubated with filipin (B), and visualized by triple fluorescence microscopy. Enlarged views of boxed areas are shown to the right of the images, and filipin staining is pseudocolored in gray. (C) FLOT2-GFP plasmid was transfected into *Anaplasma*-infected RF/6A cells (1 dpi). At ∼2 dpt, the cells were fixed, chemically permeabilized, incubated with TNM-AMCA, and analyzed by fluorescence microscopy. TNM-AMCA (blue) is pseudocolored in red. DIC, differential interference contrast of an *Anaplasma* inclusion. Images shown above are representative of at least two independent experiments. Scale bar = 5 µm. The boxed areas are enlarged to the right.

Endogenous FLOT2 has been shown to be partially colocalized with cellular cholesterol by filipin labeling in mouse oligodendroglial and human kidney epithelial cells (18, 19). To determine whether FLOT1 and FLOT2 vesicles surrounding Anaplasma inclusions contain unesterified cholesterol, triple-fluorescence analyses using FLOT2-GFP, anti-P44, and filipin in RF/6A cells were performed after fixation and chemical permeabilization. As indicated by filipin signals, FLOT2-containing vesicles docked on Anaplasma inclusions were enriched in free cholesterol (Fig. 4A). Live-cell imaging using mCherry-Anaplasma in FLOT2-GFP-transfected cells without chemical permeabilization and fixation confirmed docking of these free cholesterol-enriched FLOT2 vesicles on Anaplasma inclusions and showed stronger filipin labeling than those in fixed and permeabilized cells (Fig. 4B). Another fluorescent cholesterol probe, aminomethyl coumarin acetic acid-conjugated theonellamide (TNM-AMCA), which is much brighter and shows slower photobleaching than filipin (41), showed colocalization of cholesterol with FLOT1- and FLOT2-containing vesicles in uninfected cells (arrows in Fig. S4A and B), and the FLOT2 vesicles on Anaplasma inclusion membranes were enriched in unesterified cholesterol (Fig. 4C and S4C). Taken together, these results indicate that FLOT1 and FLOT2 vesicles docked on inclusions are enriched in unesterified cholesterol.

10.1128/mBio.02783-18.4 FIG S4 FLOT1/2 vesicles are enriched in unesterified cholesterol and encircles *A. phagocytophilum* inclusions in infected cells. (A and B) mCherry-FLOT1 and FLOT2-GFP plasmids were transfected into RF/6A cells. At 2 dpt, the cells were fixed and permeabilized by digitonin, and then cholesterol in the cells was labeled with TNM-AMCA and visualized by fluorescence microscopy under the blue channel. In the images, blue was pseudocolored in green (A) or red (B) for better visualization. The experiments shown are representative of at least two independent experiments. White arrows highlight some of FLOT1/2 and cholesterol-positive vesicles. N, nucleus. Scale bar = 5 µm. (C) FLOT2-GFP plasmids were transfected into *Anaplasma*-infected (1 dpi) RF/6A cells. At ∼2 dpt, the cells were fixed, permeabilized, and incubated with TNM-AMCA. 5C11 monoclonal antibody was used to label *Anaplasma* P44. TNM-AMCA (blue color in merged image) was pseudocolored in grey in the enlarged panels. The images shown are representative of at least two independent experiments. *Ap, Anaplasma*. Scale bar = 5 µm. Download FIG S4, TIF file, 0.8 MB. Copyright © 2019 Xiong et al. 2019 Xiong et al. This content is distributed under the terms of the Creative Commons Attribution 4.0 International license.

### Cellular cholesterol is required for FLOT1 accumulation on A. phagocytophilum inclusions.

**FIG 5:**
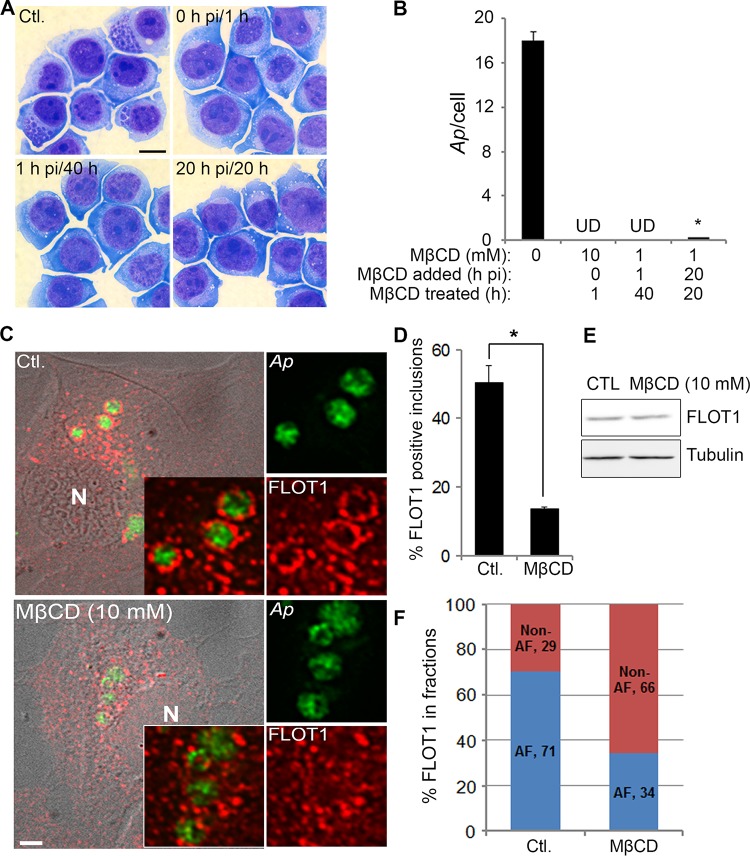
MβCD blocks *A. phagocytophilum* infection and reduces FLOT1 accumulation on *A. phagocytophilum* inclusions. (A and B) HL-60 cells were infected with host cell-free *A. phagocytophilum* (*Ap*) and at 0, 1, or 20 hpi, cells were treated with 10, 1, or 1 mM MβCD, respectively. Unbound bacteria and MβCD in 10 mM MβCD group were removed by washing at 1 hpi, whereas 1 mM MβCD remained in the medium for 20 to 40 h. In all experiments, the bacterial load was determined at 40 hpi by counting the number of Diff-Quik-stained bacteria in the cells under light microscopy. (A) Scale bar = 5 µm. The results are presented as the mean ± standard deviation from three independent experiments. (B) UD, undetectable. *, *P < *0.05. (C to E) *Anaplasma*-infected HL-60 cells treated with MβCD (10 mM) or the RPMI medium control (Ctl.) for 1 h at 37°C. (C) Cells were fixed at 24 hpi, labeled with anti-Asp62 (green) and anti-FLOT1 (red) antibodies, and visualized by DeltaVision deconvolution microscopy. N, nucleus. Scale bar = 5 µm. (D) FLOT1-enclosed inclusions in MβCD-treated and control cells were scored, and the results are presented as the mean ± standard deviation from three independent experiments. *, *P < *0.05. (E) The amount of FLOT1 protein was unchanged following MβCD treatment as determined by Western blotting using anti-FLOT1, with α-tubulin as the protein loading control (CTL). (F) *Anaplasma*-infected HL-60 cells at 2 dpi were treated with MβCD (10 mM) or the RPMI medium control for 1 h at 37°C and fractionated by density gradient ultracentrifugation (5 to 25% OptiPrep). An equal volume from each fraction was visualized by Western blotting using anti-FLOT1. Anti-P44 was used to identify fractions containing *A. phagocytophilum*. FLOT1 levels in *Anaplasma* inclusion fractions (AF, fractions 7 to 10) and non-*Anaplasma* inclusion fractions (Non-AF, fractions 1 to 6) of control and MβCD-treated cell samples were quantified based on the band intensity of FLOT1.

FLOTs are distributed in two major subcellular pools, plasma membrane and intracellular vesicular compartments, which are regulated by cellular free cholesterol (17, 19). Cholesterol depletion redistributes FLOTs to the plasma membrane, while overload of free cholesterol shifts FLOTs from the plasma membrane to intracellular compartments and leads to intracellular cholesterol accumulation in cytoplasmic vesicles (19). Methyl-β-cyclodextrin (MβCD) can be used to remove membrane cholesterol (42) by inducing cellular cholesterol efflux at 10 mM (43). We previously showed that removal of cholesterol from the membranes by pretreatment of host cell-free A. phagocytophilum or of host cells with MβCD (10 mM) for 30 min effectively abolishes the infection in host cells by blocking Anaplasma internalization (7, 11). To investigate the inhibitory effects of MβCD on Anaplasma replication, infected HL-60 cells were treated with 1 mM MβCD starting at 1 hpi (after internalization) or 20 hpi (beginning of exponential growth) for a total of 40 and 20 h of treatment, respectively (Fig. 5A and B). Both treatments almost completely abolished bacterial infection, and brief pretreatment with 10 mM MβCD at 0 hpi for 1 h inhibited bacterial internalization (Fig. 5A and B). These treatments did not reduce the viability of the host cells as shown in Fig. 5A and determined by a trypan blue exclusion assay (data not shown). The data showed that the cholesterol-disrupting drug MβCD can clear Anaplasma infection from host cells.

We further analyzed localization of FLOT1 on Anaplasma inclusions following MβCD treatment of Anaplasma*-*infected cells at 2 dpi. In control untreated cells, FLOT1 encircled bacterial inclusions and showed a punctate pattern on the inclusion membrane; however, this localization pattern was diminished in the cells treated with MβCD (10 mM) for 1 h (Fig. 5C). The percentage of FLOT1-encircled inclusions was significantly reduced by MβCD treatment (Fig. 5D), although total cellular FLOT1 levels were not changed by this brief treatment (Fig. 5E). In agreement with the morphological data (Fig. 5C and D), subcellular fractionation with an OptiPrep gradient centrifugation and Western blot analysis showed that a smaller amount of FLOT1 was cofractionated with Anaplasma inclusions (fractions 7 to 10 as shown in Fig. 3E) in MβCD-treated cells than in the control cells (Fig. 5F).

**FIG 6:**
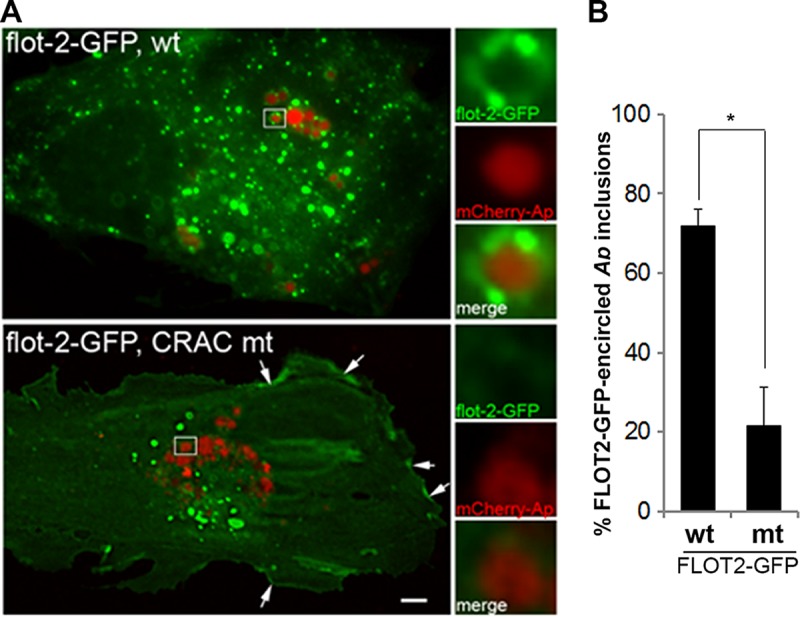
FLOT2 localization to *A. phagocytophilum* inclusions is CRAC domain dependent. Wild-type (wt) or CRAC mutant (mt) of FLOT2-GFP plasmids was transfected into mCherry-*A. phagocytophilum* (*Ap*)-infected RF/6A cells at 1 dpi. At 1 dpt/2 dpi, the cells were fixed and visualized by DeltaVision deconvolution microscopy (A). Scale bar = 5 µm. The boxed areas are enlarged to the right. (B) The percentage of cells with *Anaplasma* inclusions encircled with FLOT2-GFP was scored in >200 cells. The results are presented as the mean ± standard deviation. *, *P < *0.05.

FLOT2 has two putative cholesterol recognition/interaction motifs (CRAC) (18), which largely determine translocation of FLOT2 from the plasma membrane pool to the subcellular vesicular pool. A previous study by Strauss et al. showed that wild-type FLOT2-GFP predominantly distributes in vesicular compartments, whereas the double CRAC mutant (Y124G/Y163G) of FLOT2-GFP is retained at the plasma membrane (19). Live-cell image analysis using FLOT2-GFP wild-type and CRAC mutant-transfected RF/6A cells incubated with mCherry-A. phagocytophilum found that the proportion of inclusions encircled by FLOT2 CRAC mutant-GFP was significantly lower than with wild-type FLOT2-GFP (Fig. 6). In agreement with the findings in mouse oligodendroglial precursor cells (19), the FLOT2-GFP CRAC mutant showed more plasma membrane and less intracellular vesicular localization in transfected RF/6A cells (arrows in Fig. 6A and S5). Taken together, these data suggest that localization and accumulation of FLOTs on Anaplasma inclusions are dependent on cholesterol and the CRAC domain of FLOTs.

10.1128/mBio.02783-18.5 FIG S5 FLOT2-GFP CRAC mutant is retained at the plasma membrane. FLOT2-GFP CRAC mutant plasmid was transfected into RF/6A cells at 1 dpi. At 1 dpt/2 dpi, the cells were fixed and visualized by DeltaVision fluorescence deconvolution microscopy. Arrows indicate clear plasma membrane localization of the FLOT2-GFP CRAC mutant. The data shown are representative of at least three independent experiments. Scale bar = 5 µm. Download FIG S5, TIF file, 0.4 MB. Copyright © 2019 Xiong et al. 2019 Xiong et al. This content is distributed under the terms of the Creative Commons Attribution 4.0 International license.

### FLOT2 vesicles colocalize with DiI-LDL.

**FIG 7:**
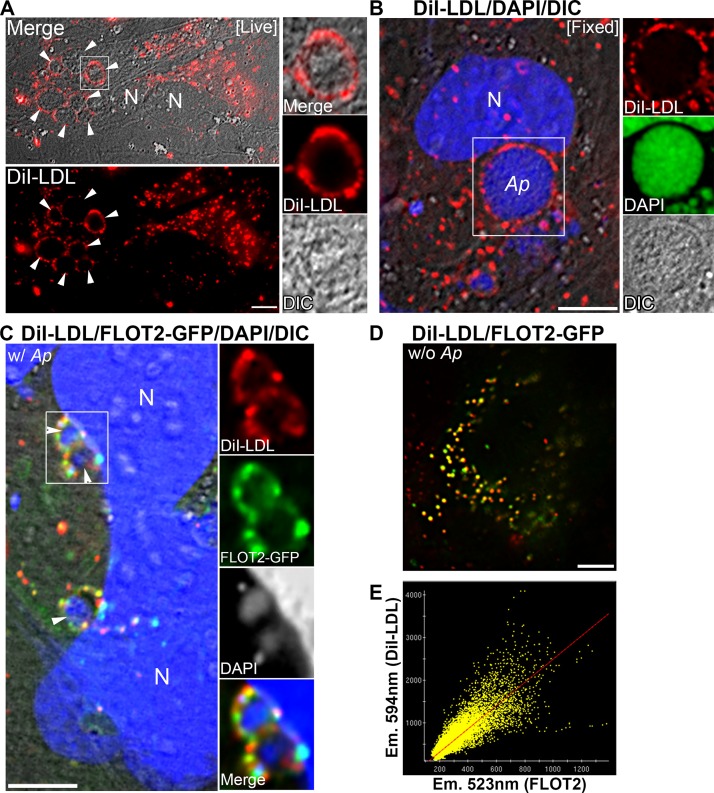
FLOT2 vesicles colocalize with DiI-LDL. (A and B) *Anaplasma*-infected RF/6A cells were preconditioned in growth medium with 10% LPDS for 12 h, and DiI-LDL was added into the cells and incubated for 3 to 5 h. The cells were immediately subjected to fluorescence microscopy without fixation ([Live]) (A) or fixed with PFA and stained with DAPI before observation ([Fixed]) (B). Arrowheads indicate *Anaplasma* inclusions, which were surrounded by DiI labeling (A). Enlarged views of boxed areas are shown to the right of the images. DAPI is pseudocolored in green in enlarged views (B). DIC, differential interference contrast of an *Anaplasma* inclusion. (C) FLOT2-GFP-transfected (3 dpt) and *Anaplasma*-infected (2 dpi) RF/6A cells were cultured in growth medium with 10% LPDS for 12 h, followed by incubation with DiI-LDL for 3 to 5 h. Cells were then fixed and stained with DAPI before microscopy observation. Enlarged views of boxed areas are shown to the right of the images. DAPI is pseudocolored in gray in enlarged views. (D and E) RF/6A cells were transfected with FLOT2-GFP and treated with DiI-LDL as described above, and they were then subjected to DeltaVision deconvolution fluorescence microscopy. The colocalization of FLOT2-GFP (emission wavelength [Em.], 523 nm) and DiI-LDL (Em., 594 nm) was analyzed in uninfected RF/6A cells, and the Pearson coefficient of correlation (*R* = 0.772 ± 0.087) was calculated using the SoftWoRx software. All figures shown above are representative of at least two independent experiments. N, nucleus. Scale bar = 5 µm.

DiI-LDL is a red florescent dye-labeled lipid-LDL complex used for studying LDL cholesterol delivery via receptor-mediated endocytosis (44). We previously showed that A. phagocytophilum enhances DiI-LDL uptake and that DiI-LDL surrounds Anaplasma inclusions in HL-60 cells (9). Here, we examined whether Anaplasma inclusion-associated FLOT vesicles contain DiI-LDL. Because cholesterol is soluble in methanol or acetone, FLOT antibody labeling which requires methanol or acetone fixation is not possible. Therefore, we performed DiI-LDL labeling in FLOT2-GFP-transfected RF/6A cells. We observed predominant DiI-LDL surrounding Anaplasma inclusions in RF/6A cells as visualized by differential interference contrast (DIC) without any fixation (Fig. 7A) or with paraformaldehyde (PFA) fixation (Fig. 7B), in agreement with our previous observation in HL-60 cells (9). In FLOT2-GFP-transfected and Anaplasma-infected RF/6A cells, FLOT2-GFP vesicles and DiI-LDL were mostly colocalized and surrounded Anaplasma inclusions (Fig. 7C). Importantly, even in uninfected cells, there was partial colocalization (Pearson coefficient of correlation = 0.772 ± 0.087) of FLOT2-GFP with DiI-LDL in transfected RF/6A cells (Fig. 7D and E), indicating that FLOT2 is associated with vesicular traffic of LDL-derived cholesterol that is acquired by A. phagocytophilum (9).

### FLOTs are required for DiI-LDL cholesterol delivery to A. phagocytophilum.

**FIG 8:**
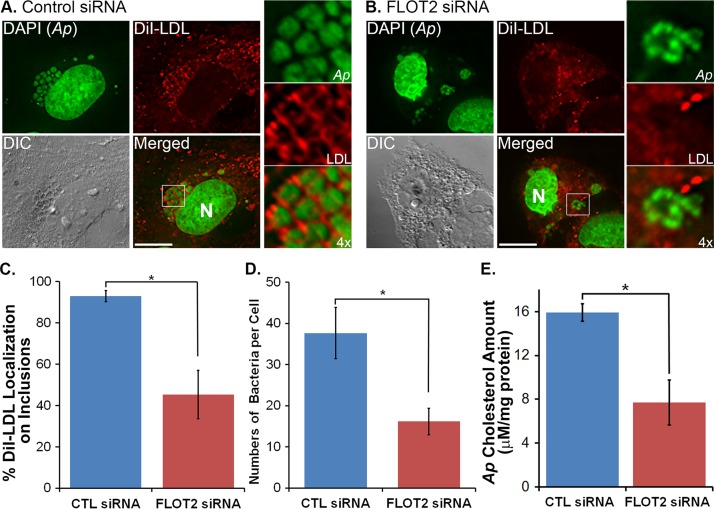
FLOT2 is required for cholesterol trafficking to *A. phagocytophilum* inclusions. (A to D) *A. phagocytophilum* (*Ap*) infection and DiI localization in *Anaplasma* inclusions were significantly reduced by FLOT2 siRNA transfection. RF/6A cells were transfected with scrambled control (CTL) or FLOT2 siRNA (1.5 μg) and infected with *A. phagocytophilum* at 1 dpt. Culture media were replaced with the growth media containing LPDS at 1 dpi, incubated for 1 day, and were labeled with DiI-LDL for 5 h. Cells were fixed with PFA, labeled with DAPI (pseudocolored in green), and observed under a DeltaVision microscope. Images were representatives from 3 independent experiments (A and B). Scale bar = 10 μm. The boxed areas are enlarged to the right. (C and D) Percentage of DiI-LDL localization of on *Anaplasma* inclusions (C) or bacterial numbers per cell (D) were quantitated from over 100 morulae from 3 independent experiments. *, P < 0.05, two-tailed *t* test. (E) FLOT2 siRNA knockdown reduced cholesterol uptake of *A. phagocytophilum*. HL-60 cells were transfected with control (CTL) or FLOT2 siRNA by the Amaxa Nucleofector system, and at 4 hpt, cells were infected with *A. phagocytophilum* and cultured for additional 2 days. Host cell-free *A. phagocytophilum* was purified, and cholesterol levels were measured using an Amplex Red cholesterol assay kit. Total cholesterol content was normalized to the amount of bacterial proteins. Results shown are the averages from 3 independent experiments. *, *P < *0.05, two-tailed *t* test.

To examine the roles of FLOTs in cholesterol delivery to A. phagocytophilum within the inclusion, RF/6A cells were first transfected with FLOT2 siRNA to knock down the protein expressions of both FLOT1 and FLOT2 (Fig. S1) and infected with A. phagocytophilum. DiI-LDL labeling and fluorescence microscopy results showed that DiI-LDL localization surrounding Anaplasma inclusions was significantly reduced by FLOT2 siRNA transfection compared to the control siRNA-transfected groups (Fig. 8A to C). In agreement with P44 protein levels shown in Fig. 1G, Anaplasma infection (by counting bacterial numbers per cell) was significantly reduced by FLOT2 siRNA transfection compared to the control groups (Fig. 8A, B, and D).

To analyze whether this reduced DiI-LDL localization to Anaplasma inclusions alters cholesterol acquisition by A. phagocytophilum. HL-60 cells were transfected with control or FLOT2 siRNA and infected with A. phagocytophilum and host cell-free Anaplasma bacteria were purified at 2 dpi. Results of an Amplex Red cholesterol assay showed that the bacterium-associated cholesterol amount was significantly reduced in FLOT2 siRNA-transfected cells compared to the control siRNA group (Fig. 8E). These results indicate that FLOTs are required for LDL-derived cholesterol trafficking to Anaplasma inclusions and cholesterol acquisition by A. phagocytophilum; therefore, FLOTs are crucial for Anaplasma infection in host cells.

### AL-containing vesicles are recruited to A. phagocytophilum inclusions and colocalized with FLOT2, whereas the AL inhibitor orlistat blocks A. phagocytophilum infection.

**FIG 9:**
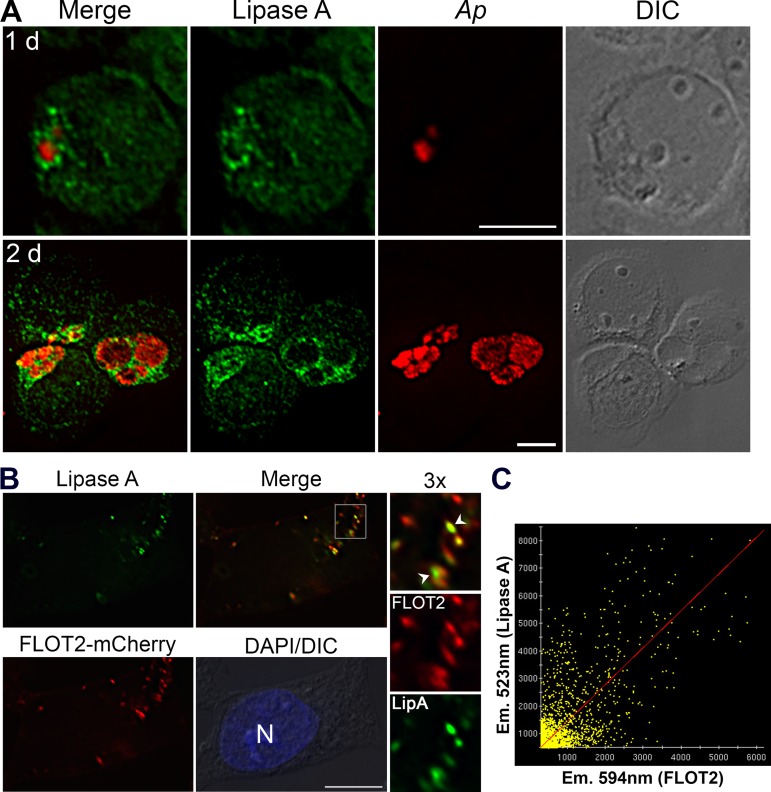
AL-containing vesicles surround *A. phagocytophilum* inclusions and colocalize with FLOT2. (A) *A. phagocytophilum* (*Ap*)-infected HL-60 cells at 1 and 2 dpi were fixed with cold methanol and labeled with anti-lipase A (green) and mouse monoclonal antibody (MAb) 5C11 (*Ap*, red). (B) HEK293 cells were electroporated with plasmids encoding FLOT2-mCherry. At 2 dpt, cells were fixed with cold methanol and labeled with anti-lipase A (green). DNA was stained by DAPI. Images were analyzed by DeltaVision deconvolution microscopy. The boxed areas are enlarged to the right. LipA, lipase A; N, nucleus; Merge, merged images; DIC, differential interference contrast. Scale bar = 5 µm. The results are representative of three independent experiments. (C) The colocalization of FLOT2-mCherry (emission wavelength [Em.], 594 nm) and lipase A (Em, 523 nm) was analyzed in transfected HEK293 cells as shown in panel B, and the Pearson coefficient of correlation (*R* = 0.796 ± 0.070) was calculated with the SoftWoRx software from over 100 cells from 3 independent experiments (a representative image was shown).

LDL is taken up via an LDL receptor by clathrin-mediated endocytosis (45), which subsequently enters an early compartment where cholesteryl ester in LDL is hydrolyzed into free cholesterol by the enzyme acid lipase (AL) (28). Because DiI-LDL and NPC1-containing vesicles were recruited onto Anaplasma inclusions (9, 10), we examined the relation between Anaplasma inclusions and this AL-containing compartment. We used an acid lipase A antibody to perform immunofluorescent labeling of Anaplasma-infected HL-60 cells and found that Anaplasma inclusions were encircled with lipase A-positive vesicles at 1 dpi and 2 dpi (Fig. 9A). As free cholesterol is liberated in acid lipase compartments from LDL cholesterol complex, FLOTs are likely recruited to this compartment. In uninfected HEK293 cells transfected with FLOT2-mCherry, immunofluorescent labeling using anti-lipase A antibody showed that most FLOT2- and lipase A-containing vesicles were colocalized (Fig. 9B and C, arrowheads; Pearson coefficient of correlation = 0.796 ± 0.070), suggesting that FLOTs play an important role in the delivery of unesterified cholesterol derived from this compartment to Anaplasma inclusions.

**FIG 10:**
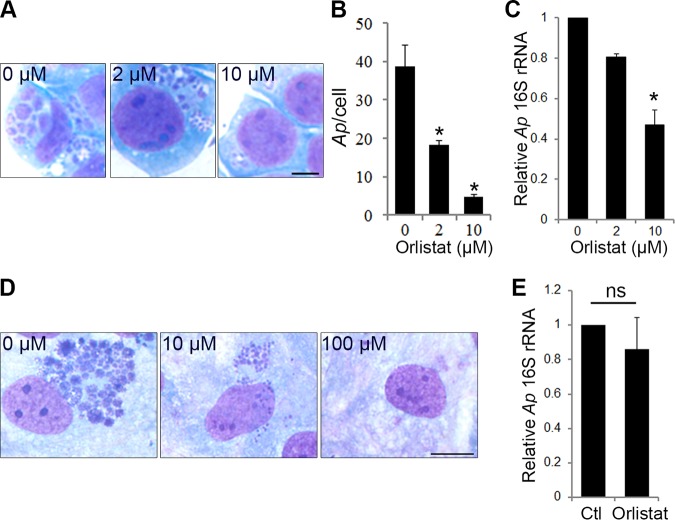
AL inhibitor orlistat inhibits *A. phagocytophilum* (*Ap*) infection in host cells. (A to C) Orlistat (2 or 10 µM) was added into *Anaplasma*-infected HL-60 at 2 hpi and kept in the medium. The bacterial infection was determined at 2 dpi by Diff-Quik staining and qPCR by detecting *Anaplasma* 16S rRNA gene normalized by human G3PDH gene. The results are presented as the mean ± standard deviation from three independent experiments, with control cells set as 1. *, *P < *0.05. (D) Orlistat (10 or 100 µM) was added into *Anaplasma*-infected RF/6A cells at 1 dpi when the inclusions were clearly seen and kept in the medium for 1 day. The infection was determined at ∼3 dpi by Diff-Quik staining. Scale bar = 5 µm. (E) Host cell-free *A. phagocytophilum* treated with orlistat (10 µM) for 30 min at 37°C were added into HL-60 cells after removal of orlistat by washing. The infection was determined at 2 dpi by qPCR by detecting the *Anaplasma* 16S rRNA gene normalized by the human G3PDH gene. The results are presented as the mean ± standard deviation from three independent experiments, with control cells set as 1. ns, no significant difference.

Orlistat, an AL inhibitor, was recently identified as a potential NPC disease therapeutic drug by small-molecule compound library screening, because orlistat can greatly lower cholesterol accumulation in NPC mutant cells by blocking AL activity (46–48). Because of the association of the AL compartment with Anaplasma inclusions and FLOT vesicles, we examined the effects of orlistat on Anaplasma infection. We found that orlistat effectively blocked Anaplasma infection in both HL-60 and RF/6A cells in a dose-dependent manner (Fig. 10A to D). Upon orlistat treatment, Anaplasma inclusions in both HL-60 and RF/6A cells were much smaller or even undetectable, indicating an arrest of bacterial growth inside the host cells, and orlistat was not toxic to the host cells at the concentrations used (Fig. 10A and D). Orlistat did not directly target A. phagocytophilum itself because pretreatment of bacteria with orlistat did not affect Anaplasma infection in the host cells (Fig. 10E). These data indicate the critical role of AL in Anaplasma infection of host cells.

## DISCUSSION

A. phagocytophilum infection upregulates LDL cholesterol uptake and is dependent on LDL-derived cholesterol, but not *de novo*-synthesized cholesterol, in the host cells for proliferation (9). Consequently, several host cell cholesterol-associated proteins in the LDL cholesterol vesicular traffic pathway, namely LDL receptor (LDLR) (9) and NPC1 (10), are required for A. phagocytophilum infection. The present study uncovered that additional cholesterol-binding host cell molecules FLOT1 and FLOT2 are required for A. phagocytophilum replication. Importantly, our result of FLOT localization to the compartment containing LDL suggests a fundamental role of FLOTs in intracellular vesicular transport of LDL-derived cholesterol in mammalian cells. To our knowledge, there has been no report on FLOT localization to the LDL compartment. One study reported that within the first 10 min following binding, LDL and transferrin, both classical ligands that induce the clathrin-mediated endocytosis, showed only 9% colocalization with FLOT1-GFP, and FLOT1 siRNA knockdown did not inhibit the internalization of transferrin, or presumably of LDL, either (49), likely because FLOT1 functions in clathrin-independent endocytosis (50). Thus, our finding suggests that FLOTs are not involved in LDLR-mediated endocytosis *per se*, but rather function downstream of endocytosis.

As FLOTs are associated with the cholesterol-rich lipid raft of various cellular membranes, including intraluminal vesicles of multivesicular bodies (19), FLOTs are involved in cellular transport of bulk cholesterol. For example, Strauss et al. (19) reported on FLOT2-dependent exosomal cholesterol secretion as a new cellular cholesterol homeostasis mechanism, and this exosomal cholesterol release is increased when cells are overloaded with free cholesterol, such as in NPC1 mutant CHO cells, in fibroblasts derived from NPC1 patients, or by treatment with an intracellular vesicular transport inhibitor of LDL-derived cholesterol, U18666A. Our data showed that cholesterol-enriched FLOT vesicles and LDL- and acid lipase-containing vesicles are targeted to A. phagocytophilum inclusions, and interfering with the targeting reduces A. phagocytophilum cholesterol amount and inhibits bacterial infection, suggesting that these vesicles are hijacked to deliver cholesterol to the inclusions. As NPC1-containing vesicles are also routed to Anaplasma inclusions and are a part of the LDL-derived cholesterol transport pathway, it is possible that these cholesterol-enriched vesicles are coordinated to deliver free cholesterol to Anaplasma inclusions. However, it is currently unknown whether the vesicles fuse with Anaplasma inclusion membranes and whether FLOTs are integrated into the inclusion membrane.

After internalization into mammalian cells, LDL enters an endosomal compartment where cholesteryl esters are hydrolyzed by AL, and then LDL cholesterol enters the NPC1-containing compartment (45). The mechanisms of cholesterol transport process among these compartments remain largely unknown. Urano et al. previously reported that *trans*-Golgi SNARE syntaxin 16 and vesicle-associated membrane protein 4 (VAMP4) are involved in vesicular trafficking of a significant portion of LDL cholesterol from the NPC1-containing endosomal compartment to the *trans*-Golgi network before reaching to the endoplasmic reticulum (ER) or plasma membrane (51). However, the mechanism of cholesterol transport from the AL-containing compartment to the NPC1 compartment is not known. Our study revealed that FLOTs are colocalized to LDL, free cholesterol, and AL-containing vesicles, suggesting that FLOTs can bind to the free cholesterol liberated from endocytosed LDL in the AL-containing vesicles. These data also suggest a potential role of FLOTs in regulating free cholesterol transport in the LDL-derived cholesterol vesicular transport pathway.

FLOT1 and FLOT2 share ∼50% amino acid sequence identity, colocalize, and coimmunoprecipitate. In fact, the major pool of cellular FLOTs appears to be hetero-oligomers (21, 33, 52). In agreement with previous reports (33–35), the expression of FLOT1 and FLOT2 is interdependent, and the depletion of one FLOT reduces the stability of the other (Fig. S1). We found that both FLOT1 and FLOT2 are localized to Anaplasma inclusions and required (directly or indirectly) for Anaplasma infection in the host cells. Thus, they may also function as a heterodimer or oligomer in Anaplasma infection.

The present study revealed FLOT/LDL vesicles are recruited to Anaplasma inclusions in a cellular cholesterol-dependent manner. FLOT1 is involved in several other intracellular bacterial infections, as FLOT1, but not FLOT2, localizes to C. pneumoniae inclusion membranes, and FLOT1 is important for C. pneumoniae intracellular growth, as determined by FLOT1 siRNA knockdown (27). FLOT1 has been found in the parasitophorous vacuole membranes of C. burnetii and M. marinum cultured in Vero cells and peripheral blood monocytes, respectively (25, 26). Infection of M. marinum that replicates in the LAMP1-positive compartment is compromised in a FLOT ortholog vacuolin B mutant of Dictyostelium amoebae, suggesting an important role of host FLOT during mycobacterial intracellular infection (26). Brucella spp. recruit FLOT1 to the vicinity of Brucella-containing vacuoles likely by secreting cyclic β-1,2-glucan, a Brucella virulence factor, which thereby appears to block lysosome fusion and allow intracellular Brucella to survive (24). However, whether cellular cholesterol is involved in FLOT recruitment has not been reported in any of these bacteria.

FLOT expression is altered under certain pathological conditions, such as neurodegenerative disorders and cancer (53). However, the regulation of FLOT expression at the protein and mRNA levels is poorly understood despite their almost ubiquitous expression and important functions in various cellular processes (39, 54). In the present study, we demonstrated that Anaplasma infection upregulated mRNA and protein amounts of FLOT1 in human leukocytes (HL-60 cells), and FLOT knockdown effectively blocks infection by the intracellular bacterium A. phagocytophilum. This study provides the first example of FLOT upregulation induced by an infectious agent. Detailed mechanisms of FLOT upregulation by A. phagocytophilum remains to be studied, but bacterial protein synthesis is clearly required.

Cyclodextrins (CDs) are a family of cyclic oligosaccharides composed of a hydrophilic outer surface and a hydrophobic core which has a high affinity for sterols. Because of these characteristics, CDs have long been used to manipulate cellular cholesterol levels, although the detailed mechanisms, particularly whether CDs directly extract cholesterol from the plasma membrane or also from organelles inside the cell, are still controversial (42). Similar to MβCD, hydroxypropyl-β-cyclodextrin (HPCD) removes the cytoplasmic cholesterol of fibroblasts at 36 mM (55). Due to the therapeutic benefit of HPCD in an NPC mouse model (56, 57), it has recently been approved for the treatment of human NPC disease, a fatal neurodegenerative lysosomal lipid storage disorder. This compound is likely effective because CDs can be delivered via pinocytosis to late endosomes/lysosomes, and by binding cholesterol, HPCD promotes cholesterol efflux out of late endosomes/lysosomes, bypassing the potential requirement for NPC2 and NPC1 for cholesterol transport (58). However, the mechanisms of CD effects, both *in vitro* and *in vivo*, are still not well understood. The present study revealed that FLOT localization to Anaplasma inclusions is sensitive to MβCD, indicating that cholesterol-enriched FLOT vesicular trafficking is dependent on free cholesterol. Our finding of MβCD, which effectively blocks Anaplasma infection, suggests that CD analogs or other lipid-raft–modifying drugs may have therapeutic potential for HGA.

Orlistat, a well-known inhibitor of various lipases, including lysosomal AL, was recently identified by using chemical library screening as one of the effective compounds in reducing the abnormal free cholesterol accumulation in lysosomes by targeting the AL enzyme that hydrolyzes LDL-derived cholesteryl esters to free cholesterol (46). Among these hit compounds, thiadiazoles containing a carbamate moiety at C-3 are effective inhibitors of lysosomal AL, and new generation of lysosomal AL inhibitors like lalistat 2 are under development for the treatment of NPC disease (47, 58). Orlistat effectively inhibits A. phagocytophilum infection in the human leukocytes, likely by targeting AL, and offers another potential therapeutic strategy for HGA.

## MATERIALS AND METHODS

### Antibodies and plasmids.

Two anti-Anaplasma antibodies were used to facilitate double-immunofluorescence labeling with other rabbit or mouse antibodies. The two antibodies are comparable in detecting Anaplasma bacteria. The rabbit anti-APH0404 (Asp62) antibody recognizes the C terminus of Anaplasma surface-exposed outer membrane protein Asp62 (59), and mouse monoclonal 5C11 recognizes the N-terminal conserved region of the Anaplasma outer membrane protein P44 (29, 30). The following antibodies were also used: mouse monoclonal anti-FLOT1 and anti-FLOT2 (BD Pharmingen, San Jose, CA), rabbit anti-lipase A (Novus Biologicals, Littleton, CO), mouse monoclonal anti-GFP (Santa Cruz Biotechnology, Santa Cruz, CA), mouse monoclonal anti-α-tubulin (Santa Cruz Biotechnology), and rabbit anti-actin (Sigma, St. Louis, MO). Normal mouse and rabbit IgGs were purchased from Santa Cruz Biotechnology. Fluorescence-conjugated secondary antibodies (Alexa Fluor 488 [AF488]-conjugated goat anti-rabbit IgG and AF555-conjugated goat anti-mouse IgG) were obtained from Life Technologies (Eugene, OR), and peroxidase-conjugated secondary antibodies were from KPL (Gaithersburg, MD). Plasmids encoding FLOT1-mCherry and FLOT2-GFP (16) and plasmids encoding the wild type or CRAC mutant of FLOT2-GFP (19) have been described. Plasmids encoding FLOT2-mCherry were constructed by Xhol and BanHI digestion and gel purification of the restiction fragment of FLOT2-GFP and ligating it into the pmCherry-N1 vector.

### Anaplasma culture.

Cultivation of A. phagocytophilum strain HZ (30) and mCherry-expressing A. phagocytophilum (61) and preparation of host cell-free A. phagocytophilum (10) were described previously. The degree of bacterial infection in host cells was assessed by Diff-Quik staining (Baxter Scientific Products, Obetz, OH), and the number of Anaplasma cells was scored in >200 host cells in triplicate culture wells, as described previously (60). Bacterial infection was assessed by Western blot analysis of the Anaplasma P44 protein amount normalized by actin or tubulin or by quantitative PCR (qPCR) of the Anaplasma 16S rRNA gene normalized by the human glyceraldehyde 3-phosphate dehydrogenase (G3PDH) gene (Table S1) (10).

10.1128/mBio.02783-18.6 TABLE S1 Primers used for qPCR. Download Table S1, DOCX file, 0.1 MB. Copyright © 2019 Xiong et al. 2019 Xiong et al. This content is distributed under the terms of the Creative Commons Attribution 4.0 International license.

### qRT-PCR.

Anaplasma*-*infected and uninfected HL-60 cells were harvested, and RNA was isolated using an RNeasy kit (Qiagen, Valencia, CA). Total RNA (2 μg) was reverse transcribed using SuperScript III reverse transcriptase (Invitrogen, Carlsbad, CA) with an oligo(dT)12-18 primer or random hexamers (Invitrogen). Quantitative PCR (20 μl) included 1 μl of cDNA (corresponding to 0.2 to 0.4 μg of total RNA), 0.25 μM each primer, and SYBR green qPCR master mix (Thermo Fisher Scientific, Waltham, MA), and the PCR was performed in the MX3000P instrument (Stratagene, La Jolla, CA). The FLOT1 and FLOT2 primer sequences are described in Table S1.

### Western blotting.

Anaplasma*-*infected and uninfected HL-60 cells (2 × 10^6^) were washed and suspended in 100 µl phosphate-buffered saline (PBS; 137 mM NaCl, 2.7 mM KCl, 10 mM Na_2_HPO_4_, and 2 mM KH_2_PO_4_ [pH 7.4]) containing freshly added protease inhibitor cocktail set III (Calbiochem, San Diego, CA) and then combined with 2× sodium dodecyl sulfate-polyacrylamide gel electrophoresis (SDS-PAGE) loading buffer (4% [wt/vol] SDS, 135 mM Tris-HCl [pH 6.8], 10% [vol/vol**]** glycerol, and 10% [vol/vol] β-mercaptoethanol).

Samples were separated by SDS-PAGE in 10% (wt/vol) polyacrylamide resolving gels and then transferred to a nitrocellulose membrane using a semidry blotter (W.E.P., Seattle, WA). The membrane was blocked in blocking buffer (5% [wt/vol] skim milk [Kroger, Cincinnati, OH], 150 mM NaCl, 50 mM Tris [pH 7.5]) and then incubated with primary antibodies (1:1,000 dilution in blocking buffer) at 4°C for 12 to 16 h and subsequently with peroxidase-conjugated secondary antibodies (1:1,000 dilution) at room temperature for 1 h. Immunoreactive bands were visualized with enhanced chemiluminescence (Thermo Fisher) using an LAS3000 image documentation system (Fujifilm Medical Systems USA, Stamford, CT), and band intensities were determined by densitometry using the Multi Gauge software (Fujifilm).

The subcellular fractionation procedure was previously described (10). Briefly, after homogenization, the lysates of Anaplasma-infected and uninfected HL-60 cells were fractionated by high-speed centrifugation in a linear OptiPrep density gradient. Equal aliquots from each fraction collected from top to bottom sequentially were subjected to immunoblotting using indicated antibodies.

### Small interfering RNA transfection.

HL-60 cells were transfected with double-stranded siRNA (3 μg/2 × 10^6^ cells) using the Amaxa Nucleofector system (kit V, program T-19; Amaxa GmbH, Cologne, Germany), as described previously (62). RF/6A cells were transfected with siRNAs using Lipofectamine 2000 (Invitrogen). Verified human-specific siRNAs targeting the genes encoding FLOT1 (sc-35391; a pool of three target-specific siRNAs) and control siRNA (#4390843; a pool of four nontargeting siRNAs) that does not target any known human genes were purchased from Santa Cruz Biotechnology and Ambion (Applied Biosystems/Ambion, Austin, TX), respectively. FLOT2 siRNAs (a pool of three target-specific siRNAs, HSS103741, HSS103742, and HSS177249) were purchased from Invitrogen. Host cell-free A. phagocytophilum was added to cells 8 to 24 h after transfection, and the cultures were then incubated for 1 to 2 days. Cells were harvested and lysed in SDS-PAGE loading buffer supplemented with protease inhibitor cocktails, and the samples were subjected to Western blotting using antibodies against FLOTs and Anaplasma P44.

### Transfection and immunofluorescence labeling.

For FLOT labeling, cells were fixed in methanol-acetone (80:20 [vol/vol]) at −20°C for 20 min and blocked in PBS containing 2% (wt/vol) bovine serum albumin (fraction V; Sigma) and 0.2% (wt/vol) gelatin for 30 min at room temperature. The cells were then incubated with mouse anti-FLOT1 or FLOT2 and rabbit anti-Asp62 antibodies in the same buffer for 1 h at 37°C, followed by incubation with AF488 anti-rabbit IgG and/or AF555 anti-mouse IgG for 1 h at room temperature. Normal mouse and rabbit antibodies were used as negative controls. For lipase A labeling, the cells were fixed with methanol at −20°C for 10 min. In some experiments, 300 nM 4′,6-diamidino-2-phenylindole (DAPI) (Sigma) was used to label A. phagocytophilum and host nuclear DNA after the completion of antibody labeling. For cholesterol labeling with filipin (Sigma), the cells were fixed with 4% paraformaldehyde (PFA) and immediately stained with 50 µg/ml filipin in PBS at room temperature for 1 h. The method using TNM-AMCA to label intracellular cholesterol was described previously (41). Briefly, the cells were fixed with 4% PFA for 15 min, followed by permeabilization with 50 µg/ml digitonin (Sigma) in PBS for 5 min. The specimens were then incubated with TNM-AMCA (1 µM) for 1 h at room temperature.

For fluorescent DiI-LDL labeling, uninfected RF/6A or Anaplasma-infected cells at 2 days postinfection (dpi) were preincubated in growth medium (Advanced Minimum Essential medium with 4mM l-glutamine) containing 10% lipoprotein-deficient serum (LPDS; Kalen Biomedical, MD) for 12 h and incubated with DiI-LDL (20 µg/ml; Life Technologies, Carlsbad, CA) for 3 to 5 h at 37°C. The cells were immediately subjected to fluorescence microscopy without fixation or were fixed with 4% PFA for 10 min and stained with DAPI.

Transfection of FLOT1-mCherry, FLOT2-GFP, or FLOT2-GFP CRAC mutant plasmids was carried out in A. phagocytophilum or mCherry-A. phagocytophilum-infected RF/6A cells at 1 dpi by using FuGene HD (Roche, Indianapolis, IN) or Lipofectamine 2000 (Invitrogen) transfection reagent according to the manufacturer’s instructions. HEK293 cells (2 × 10^6^ cells in 100 μl of Opti-MEM medium; Invitrogen) were transfected with 7 μg of FLOT2-mCherry plasmid (voltage, 100 V; capacity, 1,000; resistant, ∞) in a 0.2-cm cuvette using a Gene Pulser Xcell electroporation system (Bio-Rad, Hercules, CA). Cells were seeded onto a coverslip in a 12-well plate and cultured at 37°C.

The transfected cells were analyzed at 1 to 2 dpt/2 to 3 dpi. Cells were immediately observed without fixation for mCherry-A. phagocytophilum or fixed with 4% PFA and labeled with anti-P44 and filipin, and they were observed using the DeltaVision deconvolution microscopy system (Applied Precision, Issaquah, WA). Data were processed using SoftWoRx (Applied Precision) and Adobe Photoshop (Adobe Systems, Mountain View, CA) softwares. For internalization assays, two labeling steps of 4% PFA-fixed cells with mouse anti-P44 were carried out, as follows: the first labeling step was performed without saponin permeabilization using AF555-conjugated goat anti-mouse IgG, and the second labeling step was performed using AF488 goat anti-mouse IgG after permeabilization with saponin (38). Only internalized bacteria are colored in green, and they were quantified in >200 host cells under the DeltaVision microscope.

### Chemical treatment.

MβCD (Sigma) or orlistat (Cayman Chemical, Ann Arbor, MI) was added to the Anaplasma-infected cells at the indicated time postinfection and was retained in the growth medium throughout the incubation period or was removed at the time points as indicated in the figures and figure legends. Treatments at the concentrations used did not affect host cell viability and growth as assessed by trypan blue staining and light microscopy. For some experiments, host cell-free A. phagocytophilum purified from infected HL-60 was pretreated with the chemical in growth medium without fetal bovine serum (FBS) for 30 min at 37°C. The chemical was then removed by washing, and the bacteria were incubated with host cells. The degree of bacterial infection in host cells was assessed as described above.

### Cholesterol assay of purified A. phagocytophilum.

HL-60 cells (1 × 10^6^ cells) were transfected with control or FLOT2 siRNA (3.0 μg) using the Amaxa cell kit V with program T-19 (Lonza Bioscience, Cologne, Germany) and cultured in T-25 flasks. At 4 hpt, cells were infected with A. phagocytophilum and cultured for an additional 2 days. Host cell-free A. phagocytophilum was purified by sonication and lysed in 90 μl of RIPA buffer (50 mM HEPES, 150 mM NaCl, 1% NP-40, 0.25% deoxycholate, 0.1% SDS, and protease/phosphatase inhibitors). Cholesterol levels of purified A. phagocytophilum were measured using an Amplex Red cholesterol assay kit (Invitrogen), as described previously (7, 10), and total cholesterol content was normalized to the amount of bacterial proteins detected by a bicinchoninic acid (BCA) protein assay (Thermo Fisher), as previously described (7, 9).

### Statistical analysis.

Statistical analysis was performed by a 2-tailed Student *t* test, and a *P *value of *<*0.05 was considered significant. For experiments involving more than two groups, analysis of variance was performed, and a *P *value of *<*0.05 was considered significant. All statistical analyses were performed using Microsoft Excel 2010.
