# Supplementary figures and images for: Infection by Anaplasma phagocytophilum Requires Recruitment of Low-Density Lipoprotein Cholesterol by Flotillins
Source: mBio. 2019 Mar 26;10(2):e02783-18. doi: 10.1128/mBio.02783-18 (PMC6437059; doi:10.1128/mBio.02783-18)

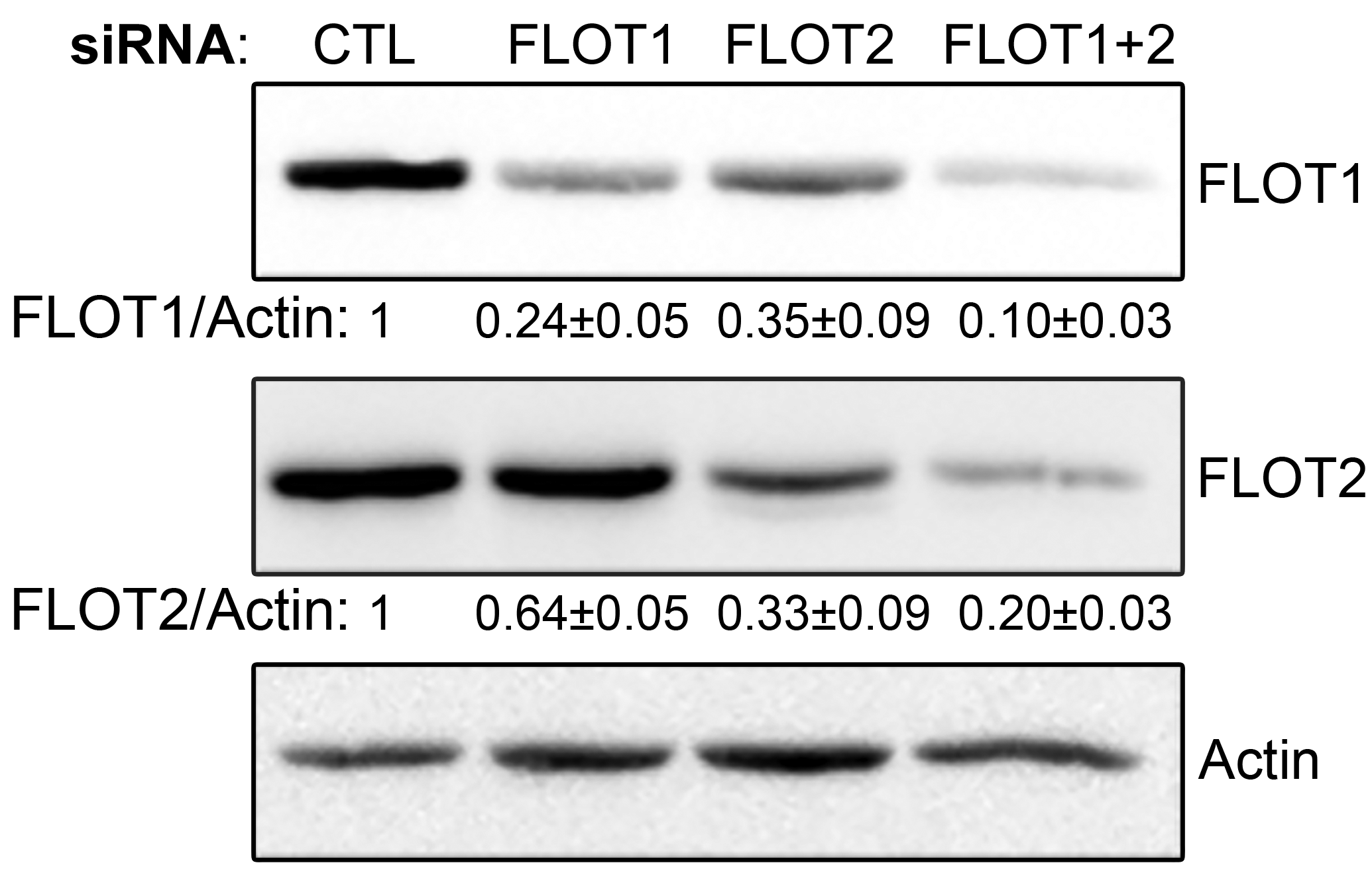

Supplement: FIG S1 [file mBio.02783-18-sf001.tif]

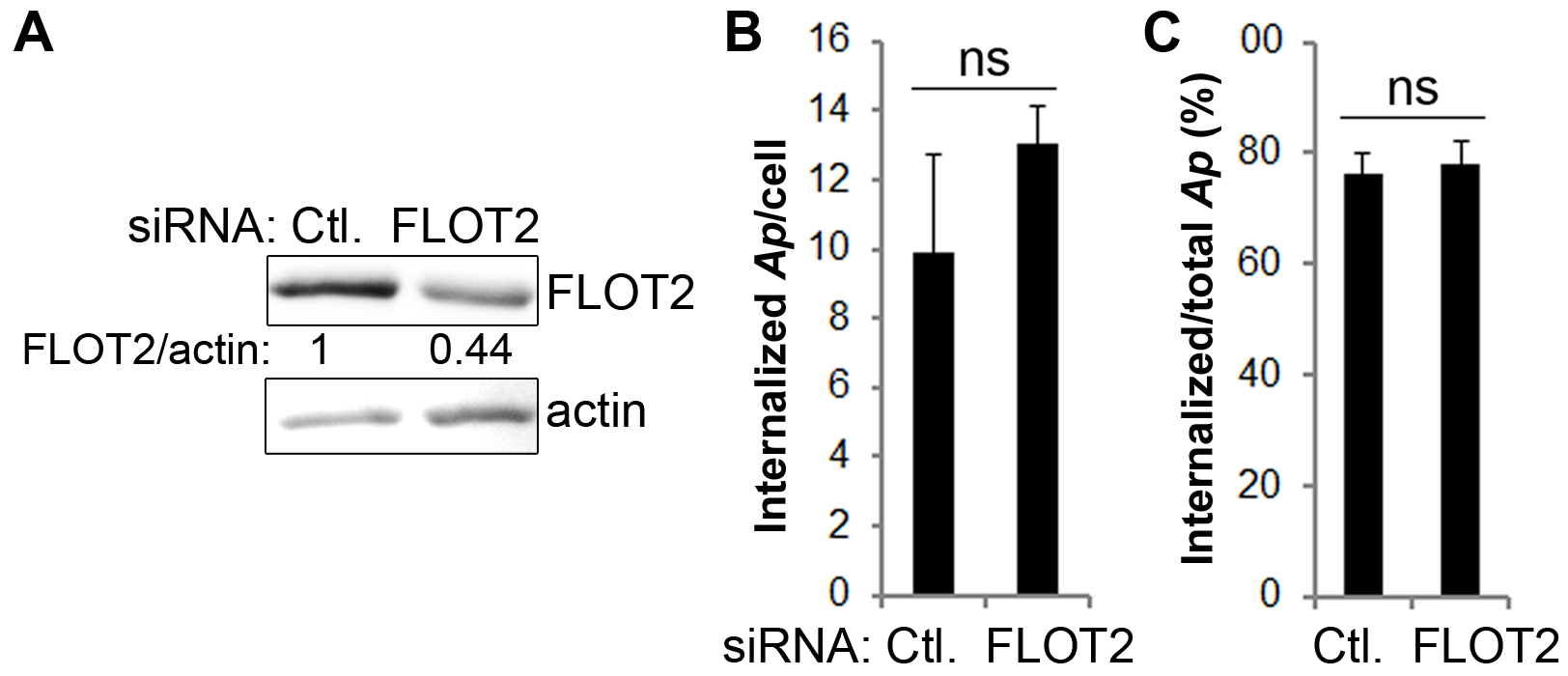

Supplement: FIG S2 [file mBio.02783-18-sf002.tif]

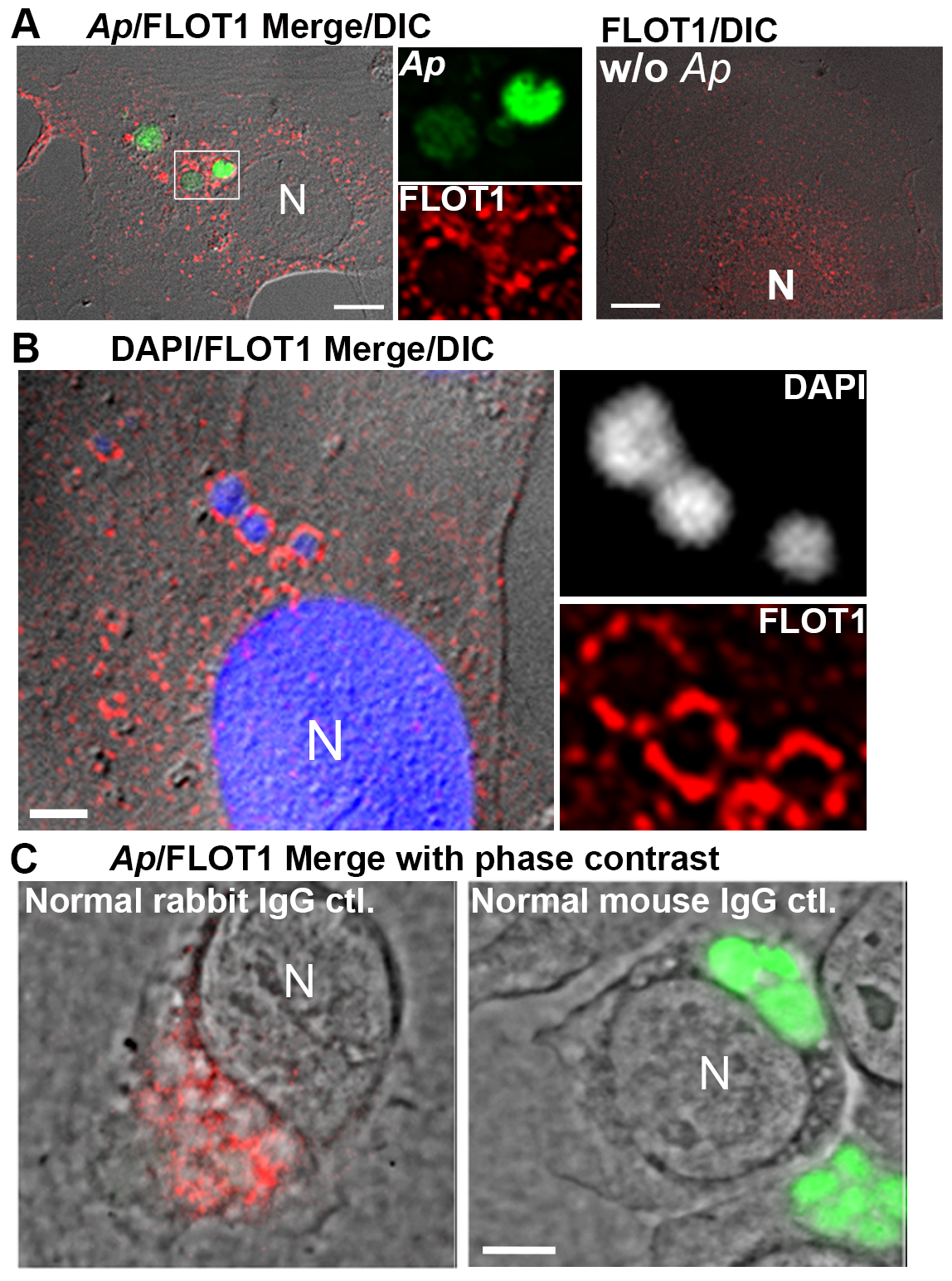

Supplement: FIG S3 [file mBio.02783-18-sf003.tif]

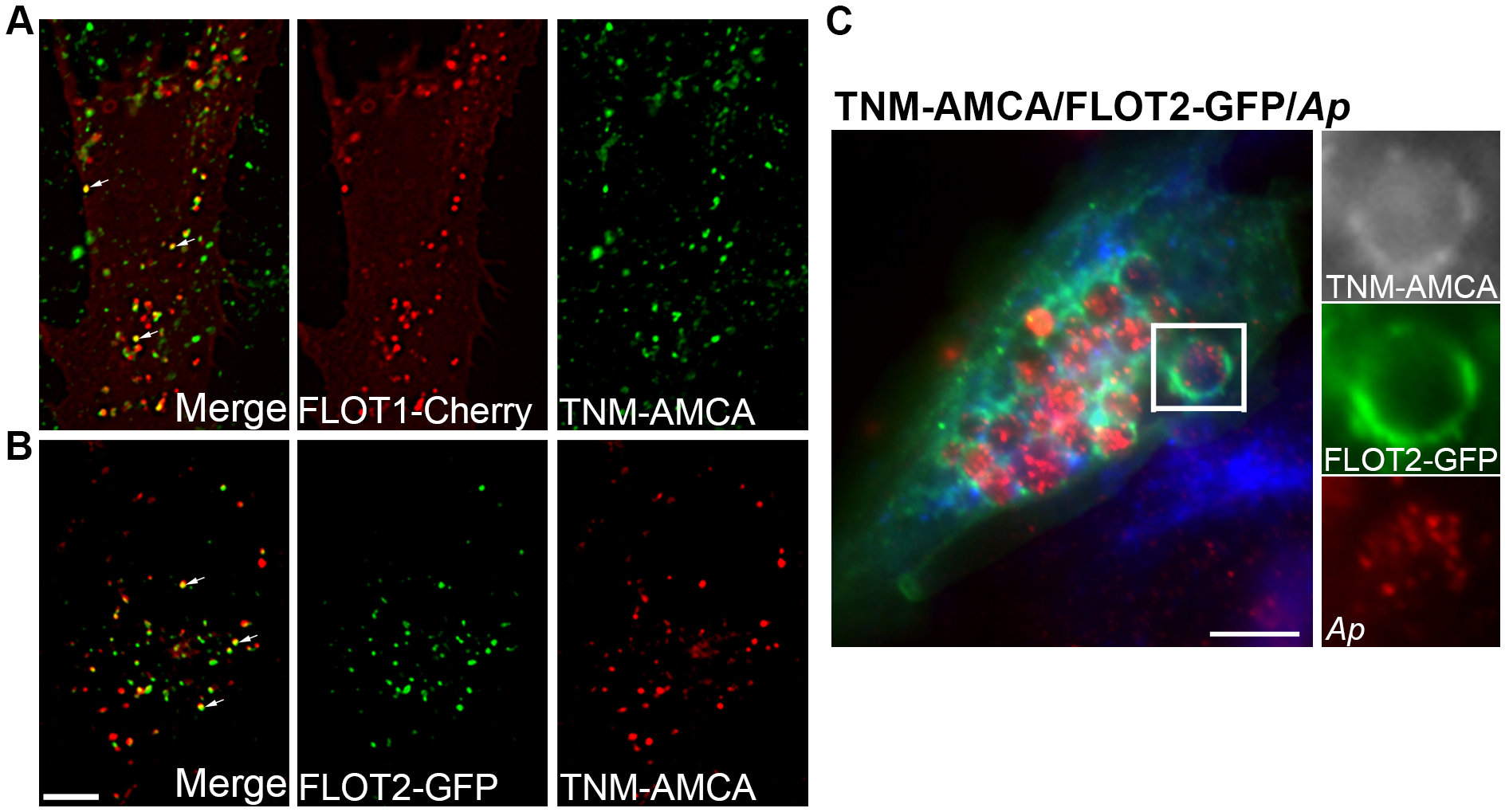

Supplement: FIG S4 [file mBio.02783-18-sf004.tif]

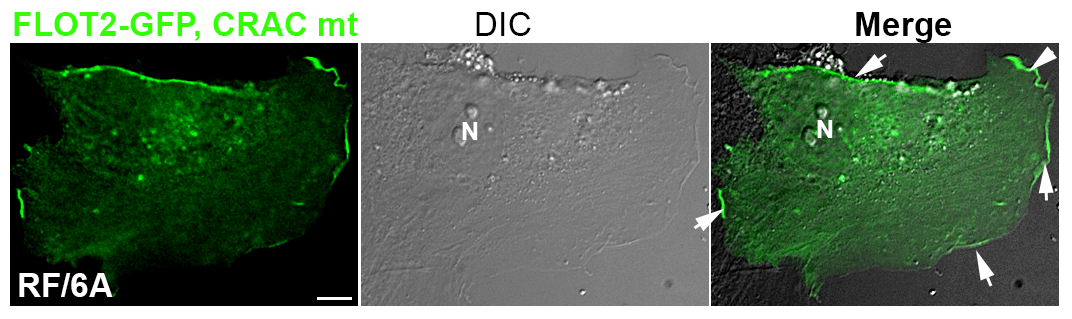

Supplement: FIG S5 [file mBio.02783-18-sf005.tif]
