# Supplementary material for: Infection by Anaplasma phagocytophilum Requires Recruitment of Low-Density Lipoprotein Cholesterol by Flotillins
Source: mBio. 2019 Mar 26;10(2):e02783-18. doi: 10.1128/mBio.02783-18 (PMC6437059; doi:10.1128/mBio.02783-18)
Supplement: TABLE S1 [file mBio.02783-18-st001.docx]

**Table S1.** Primers used for qPCR.

| **Primer ID** | **Sequence** | **Product Length** |
| --- | --- | --- |
| FLOT1 | Forward: 5’-GGGGGCAGCCAAAGTGAC-3’  Reverse: 5’-TGAGGCTGGGCATCTGTGA-3’ | 151 bp |
| FLOT2 | Forward: 5’-GCCTGGGCCTGGTGGTGTAT-3’  Reverse: 5’-TCTGCAGGACGACGTTTTGATG-3’ | 220 bp |
| *A. phagocytophilum* 16S rRNA | Forward: 5’-GGTGAGTAATGCATAGGAATC-3’  Reverse: 5’-GCTCATCTAATAGCGATAAATC-3’ | 108 bp |
| Human GAPDH | Forward: 5’-AGCAATGCCTCCTGCACCACCAAC-3’  Reverse: 5’-CCACATCACCCCTCTACCTC-3’ | 244 bp |
